# Short-Term Fasting Synergizes with Solid Cancer Therapy by Boosting Antitumor Immunity

**DOI:** 10.3390/cancers14061390

**Published:** 2022-03-09

**Authors:** Nadia de Gruil, Hanno Pijl, Sjoerd H. van der Burg, Judith R. Kroep

**Affiliations:** 1 Department of Medical Oncology, Leiden University Medical Center, 2333 ZA Leiden, The Netherlands; n.de_gruil@lumc.nl (N.d.G.); s.h.van_der_burg@lumc.nl (S.H.v.d.B.); 2 Division of Endocrinology, Leiden University Medical Center, 2333 ZA Leiden, The Netherlands; h.pijl@lumc.nl

**Keywords:** short-term fasting, fasting mimicking diet, cancer therapy, immunomodulation, cancer immunity, chemotherapy, immunotherapy

## Abstract

**Simple Summary:**

Stimulating our body’s own immune response to fight cancer is important for the success of cancer treatment in general. To further improve current cancer therapy, preclinical research shows that short-term fasting diets enhance cancer therapy efficacy, such as chemotherapy. Short-term fasting diets are low-caloric and low in protein for 3–5 days; they are usually done every couple of weeks. This review summarizes preclinical and clinical evidence of fasting diets synergizing with cancer therapy by boosting antitumor immunity.

**Abstract:**

Short-term fasting (STF), using a low caloric, low protein fasting mimicking diet (FMD), appears to be a promising strategy to enhance chemotherapy-based cancer efficacy, while potentially alleviating toxicity. Preclinical results suggest that enhanced tumor immunity and decreased growth signaling, via lowering of circulating insulin and insulin growth factor 1 (IGF-1) levels form the potential underlying mechanisms. STF may boost anti-tumor responses by promoting tumor immunogenicity and decreasing local immunosuppression. These findings warrant further studies focused on the combination of STF, not only with chemotherapy, but also with immunotherapy to evaluate the full range of benefits of STF in cancer treatment. Here, we delineate the underlying anticancer mechanisms of fasting. We summarize preclinical evidence of STF boosting antitumor immunity and alleviating immunosuppression, as well as the clinical findings reporting the immunomodulatory effects of STF during various cancer treatments, including immunotherapy.

## 1. Introduction

The nature of the local immune response is strongly associated with the success of different cancer treatments [1,2]. Short-term fasting (STF) as an adjunct to cancer treatment can reinforce tumor immunity and, therefore, boost the efficacy of chemo- and immunotherapy. The potential underlying mechanisms are increased immunogenicity of the tumor and alleviated tumor- and/or chemotherapy-induced immunosuppression [3,4]. Pre-clinical studies have well established the benefits of adding fasting to cancer therapy [5]. These include the protection of healthy cells from chemotoxicity, while sensitizing tumor cells to chemotherapy at the same time, resulting in delayed progression [3,5,6]. An increasing number of clinical trials provide evidence for benefits of STF in the treatment of cancer, including the reduction of chemotoxicity [6,7,8,9]. In recent studies, the anticancer effects of fasting were also attributed to improved antitumor immunity [3,4,10,11]. These findings suggest that fasting can reinforce the antitumor response when combined with chemotherapy, and encourage further exploration of combinations with other treatments, immunotherapy for instance.

Over the last century, preclinical studies have clearly documented the health benefits of continuous restriction of daily caloric intake by 30–60% compared to ad libitum intake [12,13]. However, continuous calorie restriction is obviously difficult to sustain. That is why current research focuses on the biological and clinical effects of STF as a more feasible alternative. Water-only fasting is safe for many weeks in healthy people [6,14], but is arguably even more difficult than continuous calorie restriction. Therefore fasting mimicking diets (FMDs) were designed to mimic its molecular physiological effects, while still allowing the consumption of (low-calorie) food, primarily comprising of complex carbohydrates and healthy fatty acids [15]. FMDs are low-caloric and low-protein diets comprising roughly 200–1100 kcal per day for the duration of 3–5 days, often repeated in cycles every couple of weeks. Intermittent fasting may be defined as short (1–2 days) periods of fasting or low caloric intake separated by 1–5 days of usual food consumption [12]. Periodic fasting is usually defined as 4 or more consecutive days of low-calorie intake, separated by weeks to months of regular consumption. Time restricted eating entails restriction of eating for a time window of roughly between 6 and 10 h a day. Time restricted eating is not primarily meant to restrict intake and can be isocaloric too [16]. Dietary restriction refers to the reduction or omission of specific groups of nutrients. Finally, calorie restriction mimetics are worth mentioning since these drugs are designed to mimic the caloric restriction effects pharmacologically while still allowing regular diet consumption [17]. Here, we mainly focus on studies evaluating the effects of intermittent/periodic fasting and FMD, but sometimes refer to results from other fasting modules. It is important to note that different fasting diets might have distinct effects in the context of cancer or antitumor immunity.

Antitumor immunity is known to be crucial in the success of any cancer treatment [1,2]. It is determined by the balance between immunogenicity and immune evasion by tumor cells [3,18]. At the start of neoplastic transformation, cancer cells elicit an immune response, driven by cytotoxic cells of the innate immune system, such as natural killer cells, neutrophils, and cytotoxic macrophages, as well as by cytotoxic CD8+ T cells from the adaptive immune system. CD4+ T cell play a supportive role in the cytotoxic attack. Unfortunately, less immunogenic cancer cells survive and are positively selected, also known as clonal selection or evolution. As the tumor progresses and evolves, more complex immune evasion maneuvers occur, involving corruption of the surrounding stromal tissue to induce local immunosuppression in the tumor microenvironment.

This narrative review will delineate on the proposed underlying mechanisms of fasting. Next, preclinical evidence to support that STF promotes antitumor immunity and alleviates immunosuppression will be summarized, as well as clinical findings on STF induced immunomodulation during various cancer treatments, including immunotherapy. Lastly, obstacles, current gaps in the literature, and future directions of research will be discussed.

## 2. Methods

**Figure 1:**
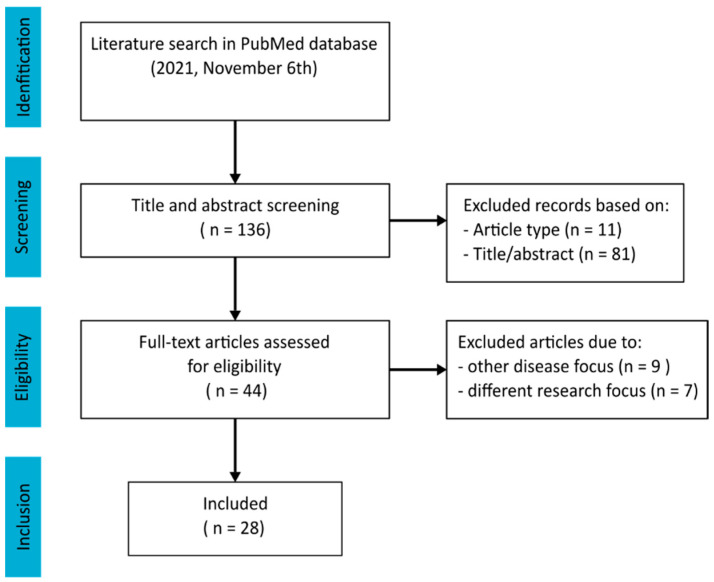
Flow diagram of article selection.

A literature search was performed in the PubMed database to systematically collect literature for this narrative review, which yielded 136 results on 6 November 2021, and was routinely checked until 10 December 2021. The terms fasting, caloric restriction, immune system phenomena, immune modulation, and neoplasm were used among others, as shown in the search strategy displayed in Supplementary File S1. At first, selection was based on title screening, abstract information, and type of publication. The articles were included if cancer and cancer therapy were the main focus. As a result, 28 articles were selected as summarized in Figure 1. Relevant references from these articles were collected using the snowballing method.

### 2.1. Fasting Causes Metabolic Reprogramming, Stimulates Autophagy, and Can Increase Immunogenic Cell Death

Fasting causes nutrient shortage on a systemic level, which results in metabolic reprogramming and induces autophagy on a cellular level via nutrient sensing pathways [15]. Interestingly, fasting differentially affects healthy cells compared to tumor cells. On the one hand, fasting increases the resistance of healthy cells to stressors, a phenomenon called differential stress resistance (DSR) [6,7,19]. On the other hand, it sensitizes tumor cells to stressors, known as differential stress sensitization [5,7,19]. As a result, fasting can lead to a form of cell death known as immunogenic cell death (ICD) in response to chemotherapy or radiation [5,20].

ICD is a specific type of cell death that often evokes an antitumor immune response [21]. Some chemotherapies are known to induce ICD, such as mitoxantrone and oxaliplatin [22,23]. During ICD, tumor cells secrete adenosine 5′-triphosphate (ATP) and calreticulin among other surface proteins [24]. ATP in particular promotes recruitment of dendritic cells (DCs) to the tumor bed [24] and calreticulin stimulates tumor antigen absorption by DCs [21]. Consequently, increased DC infiltration can lead to increased antigen presentation and subsequently elicit a tumor specific immune response.

STF induces metabolic reprogramming by systemically lowering several nutrient and hormone plasma concentrations, such as glucose, insulin, and insulin growth factor 1 (IGF-1). Directly downstream of the IGF-1 receptor (IGF-1R), the nutrient sensing pathway mammalian target of rapamycin (mTOR) integrates cell growth signals with metabolic and environmental cues via phosphoinositide 3-kinase (PI3K) and protein kinase B (Akt or AKT) [15,25,26,27]. Therefore, decreased IGF-1 plasma concentration downregulates IGF-1R–AKT–mTOR and cyclic adenosine monophosphate protein kinase A (cAMP–PKA) signaling. Decreased mTOR pathway signaling warns the cell about resource shortages and induces metabolic reprogramming to survive by lowering metabolic demand and switching cell metabolism towards other energy sources, such as fatty acids [5,19]. Lowered metabolic and mitotic activity can potentially hamper cellular uptake of chemotherapeutic toxins and limit intracellular damage induction in healthy cells [5,19]. It is this crucial metabolic switch through which fasting affects tumor cells and healthy cells differentially.

Tumor cells are less able to adapt to STF induced nutrient shortage [5,19]. This is because most tumor cells rely on anaerobic glycolysis for ATP production, known as the Warburg effect [28,29]. This increased metabolic state requires abundant energy and building blocks in the form of glucose and glutamine to support tumor growth and proliferation among other tumorigenic properties [30]. Even when oxygen is available and their mitochondria are functional, tumor cells do not use mitochondrial oxidative phosphorylation to harvest energy in the form of ATP [29]. STF appears to induce an anti-Warburg effect in tumor cells, characterized by a switch to oxidative phosphorylation and increased oxygen consumption [26,31]. During this anti-Warburg effect, some studies observed increased accumulation of reactive oxygen species (ROS) and apoptosis [31]. Indeed, STF renders tumor cells more vulnerable to other stressors, such as chemotherapy [5,19].

Healthy cells are metabolically rewired upon fasting to induce autophagy, stimulate internal repair processes, and provide protection against toxic effects of chemotherapy [6,7,19,21,26,32]. STF induces autophagy at least partially through mTOR downregulation. Autophagy is a lysosomal degradation process that plays an important role in cell survival in response to virtually any kind of stress, including starvation. It eliminates damaged organelles, misfolded proteins and invading pathogens. The breakdown products can be used as fuel and/or building blocks for starved cells [15,26]. Autophagy also forces the cell to prioritize internal repair mechanisms and it may prevent malignant transformation in the long run [15,26,33]. On top of that, autophagy balances the production of ROS, contributing to cell protection against damage by free radicals [26]. Interestingly, a growing body of evidence suggests that autophagy has a substantial role in the extension of a life- and health span induced by caloric restriction [33].

STF directly induces autophagy in tumor cells as well [26]. The role of autophagy in cancer is complex. It sometimes stimulates tumor progression, depending on the type and stage of tumor development [34]. In contrast, there is also evidence that autophagy can inhibit tumor growth, partially by depleting tumor cells of acetyl-CoA and ATP [17], which renders them more vulnerable to chemotherapy [35]. Moreover, perhaps even more importantly, autophagy contributes to ICD [5,7,21,24]. Thus, the role of autophagy in cancer (treatment) is complex and remains to be fully elucidated [21,34,35].

In summary, STF can protect healthy cells against side effects of chemotherapy, while simultaneously sensitizing tumor cells to cancer therapy. These benefits are mainly achieved through metabolic reprogramming and stimulating autophagy. Consequently, there is increased ICD of tumor cells, which may result in enhanced antitumor immunity.

### 2.2. Pre-Clinical Evidence Shows That STF Can Decrease Immunosuppression and Boost Antitumor Immunity

**Figure 2:**
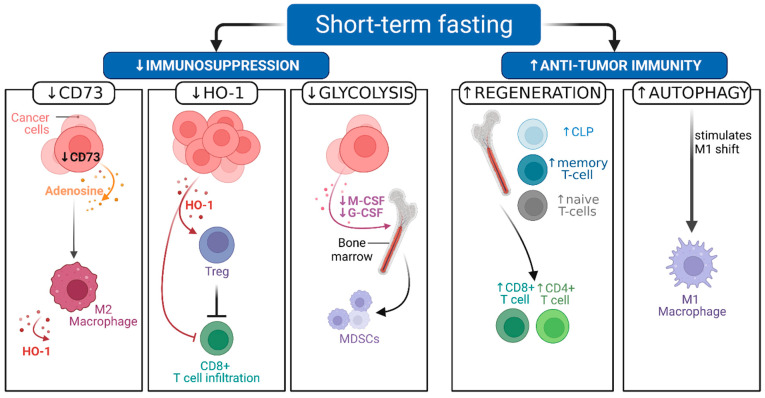
Immunomodulatory mechanisms of short-term fasting [STF] during anticancer therapy. STF reduces immunosuppression and enhances antitumor immunity via the following mechanisms established from preclinical studies: CD73 downregulation in cancer cells causes decreased adenosine release, which in turn diminishes immunosuppressive M2-type macrophage polarization. Decreased heme oxygenase-1 (HO-1) production by cancer cells (and M2 macrophages) releases inhibition of regulatory T (Tregs) cells on CD8+ cytotoxic T cells directly as well as direct inhibition from HO-1. Lowered glycolysis inhibits macrophage and granulocyte colony-stimulating factor (M-CSF, G-CSF) secretion by cancer cells. Consequently less myeloid derived suppressor cells are mobilized from the bone marrow. Hematopoietic stem cell regeneration of common lymphoid progenitors (CLP), naïve T cells and accumulation of memory T cells is observed centrally. Peripheral increase of CD8+ and CD4+ T cells is observed after refeeding and might replenish exhausted T cells as well as increase tumor antigen recognition chance. Autophagy induction stimulates tumoricidal M1 macrophage differentiation, which can support antitumor immunity. Figure 2 is adapted from “Cancer Immunoediting”, by BioRender.com (2022). Retrieved from https://app.biorender.com/biorender-templates.

Several preclinical studies have investigated the immunomodulating mechanisms of fasting, using different fasting diets, as shown in Table 1 [3,4,36]. Here, we will focus on T cells and macrophages, and we elaborate on how fasting affects their metabolism, decreases immunosuppression, promotes regeneration, and ultimately boosts antitumor immunity, as illustrated in Figure 2.

T cell metabolism is affected by STF in various short- and long-term ways. Short-term STF has negative effects on effector T cells, as they rely on glycolysis for their high metabolic demand. Consequently, effector T cells have a harder time competing with cancer cells in the tumor microenvironment for nutrients during STF [41]. Low glucose conditions are linked to increased transcription of genes that render T cells dysfunctional [42]. Nevertheless, T cells with increased glycolytic activity characterized by heightened mTOR pathway signaling can show low persistence, antigen recall response, and proliferative capacity [39,40,41]. Conversely, inhibiting mTOR signaling with rapamycin was shown to increase T cell persistence [43].

In the long-term, CD8+ and memory T cells can adapt to nutrient restriction [44]. STF increases adenosine monophosphate-activated protein kinase (AMPK) pathway activity, which can promote long-term survival and lineage stability through fatty acid oxidation [44,45]. Furthermore T cells with low metabolic activity show increased persistence and increased proliferative capacity later on [46]. Moreover, Sukumar et al. showed that T cells with low metabolic activity, used in adoptive transfer therapy of a melanoma mouse model, mediate a better antitumor response, evidenced by increased T cell proliferation and better tumor control among other things [47]. Therefore, fasting diets pose an interesting way of reducing mTOR- and increasing AMPK signaling to improve T cell mediated antitumor responses in the long term.

In addition, effector T cells experience less immunosuppression by regulatory T cells (Tregs) during STF as shown in Figure 2 [48]. A preclinical study observed Treg depletion (CD4+, CD25+, Foxp3+) during calorie restriction mimetics (hydroxycitrate, an ATP citrate lyase inhibitor) in an autophagy-competent lung cancer (TC-1 non-small cell) mouse model treated with ICD-inducing chemotherapy (mitoxantrone or oxaliplatin) [3]. Moreover, this combination treatment improved tumor growth control in a colorectal cancer and a MCA205 fibrosarcoma model as well. Furthermore, this compound effect required autophagy induction and was mediated by depleting Tregs frequency [3]. Interestingly, the hydroxycitrate treated mice showed levels of autophagy induction similar to 48 h fasted mice [3]. Contrastingly the hydroxycitrate and cisplatin combination did not show improved outcomes in the MCA205 fibrosarcoma mouse model, perhaps because cisplatin elicits ICD only minimally [3,49]. Together, these results suggest that both STF and hydroxycitrate synergize with ICD-inducing chemotherapy to improve efficacy compared to single-agent or STF treatment alone.

Another mice study by Di Biase et al. showed a reduction in intra-tumoral Tregs in B16 melanoma and 4T1 breast cancer models after 4 days FMD every 2 weeks for 2–3 cycles in addition to chemotherapy [4]. This led to an increase of CD8+ tumor infiltrating lymphocytes (TILs) and cytotoxicity in the tumor bed illustrated in Figure 2 and correlated with delayed tumor progression. Further analysis showed that the improved CD8+ infiltration and cytotoxicity was dependent on heme oxygenase-1 (HO-1) downregulation. HO-1 is an enzyme involved in the breakdown of free heme [50]. HO-1 is often described to modulate the tumor microenvironment in favor of tumor progression [51]. It is noteworthy that among other cells, Treg cells can express HO-1 to dampen T cell antitumor immunity [52], which makes HO-1 an interesting potential target for therapy.

Furthermore, Di Biase et al. found that T cells experience hematopoietic regeneration upon STF. The authors observed an increase in the common lymphoid progenitor count in the bone marrow accompanied by increased circulating naïve CD3+/CD8+ cells upon FMD [4]. This effect could theoretically aid specific antitumor T cell mediated response [4], as this new pool of T cells may respond to previous or newly formed tumor antigens. Takakuwa et al. also observed an increase in naïve T cells (CD44−, CD8+, CD4+) in the bone marrow of mice after 48 h of fasting [37]. Other investigators, using caloric restriction of 50% for 6 weeks continuously in mice models, reported remodeling of the bone marrow compartment as well [38], specifically accumulation of memory T cells. A reduction in CD4+ and CD8+ T cells within the peripheral blood and the secondary lymphoid organs was observed as well. Lymphocyte redistribution commenced after 1 week of caloric restriction and remained stable for at least 6 weeks. CD4+ and CD8+ T cell numbers significantly increased in bone marrow after 3 weeks of caloric restriction. The central memory T cell accumulation in bone marrow during caloric restriction was associated with enhanced tumor immunity and survival benefit in mice receiving melanoma-specific CD8+ T cells after a melanoma tumor challenge at 3 weeks of energy restriction [38].

Additionally, Cheng et al. observed hematopoietic stem cell regeneration effects in mice that fasted for 48–120 h around cyclophosphamide chemotherapy for six cycles in a span of 12–14 days. The hematopoietic stem cell regeneration was dependent on downregulation of IGF-1/PKA signaling, an important endocrine sequel of fasting [39]. Moreover, mortality due to chemotherapy was significantly reduced (*p* < 0.01) in mice fasted for 48 h (*n* = 10) compared to ad libitum fed mice (*n* = 10).

In contrast, the tumor promoting myeloid derived suppressor cells (MDSCs) do not benefit from hematopoietic regeneration [40]. One study showed that the MDSC (CD11b+, Gr1+) frequency was similar in peripheral blood mononuclear cells (PBMCs) but reduced in the spleen after two cycles of 4-day FMD (with 50% or 70% calorie restriction) followed by a 10-day ad libitum diet in a 4T1 breast cancer mouse model [40]. Moreover a significant reduction of tumor-resident MDSCs and an increased T cell to MDSC ratio in PBMCs was detected [53]. Similar results were achieved in a study using intermittent fasting in 4T1 and 4T07 preclinical tumor models, where decreased accumulation of granulocytic MDSCs in the spleen was associated with reduced tumor growth [54]. Another study showed that glycolysis reduction by 2-deoxy-D-glucose inhibited granulocyte- and macrophage colony-stimulating factor production by tumor cells, which resulted in less mobilization of MDSC in the 4T1 preclinical model as visualized in Figure 2 [55]. This effect was associated with improved T cell immunity, tumor growth inhibition, and prolonged survival [55].

After MDSCs are mobilized by tumor cells, they can differentiate into the heterogenous group of tumor associated macrophages (TAMs) [56]. During FMD, these macrophages differentiate less effectively into an M2 phenotype, which are immunosuppressive TAMs [36,57]. A study in colorectal cancer models demonstrated that upon FMD, CD73 expression by tumor cells is reduced and this blunts adenosine release into the extracellular environment, thereby preventing the JAK1/STAT3 signaling-dependent shift towards M2 macrophages [36]. The polarization of macrophages towards a M1-tumoricidal phenotype rather than a M2-immunosuppressive phenotype might be mediated through autophagy [58]. Fasting can induce autophagy (at least partially) via reduction of mTOR signaling. Therefore, FMD-induced downregulation of mTOR could partially alleviate TAM-driven immunosuppression in a similar way as mTOR and PI3K targeting agents are able to modulate macrophage induced immunosuppression [57,59]. Modulation of TAMs by fasting through these mechanisms is an interesting strategy, since the tumor microenvironment comprises up to 50% of TAMs in some solid tumors and high levels of TAMs are associated with adverse prognosis [57,60].

Interestingly, some of the M2-like TAMs display an increased expression of HO-1 that can dampen antitumor immunity [50,61,62]. High levels of HO-1 expression in tumor or stromal cells is associated with anti-apoptotic effects as well as therapy resistance and poor prognosis [63]. Preclinical studies have explored targeting HO-1 in anticancer therapy. For instance, Alaluf et al. showed that myeloid specific HO-1 deletion lead to enhanced antigen specific CD8+ cytotoxicity upon therapeutic immunization in the thymoma (EG7/OVA) mouse model [50]. Another study shows that the use of tin mesoporphyrin to inhibit HO-1 expression in immunosuppressive TAMs (fibroblast activation protein alpha+) in tumor bearing mice (LL2/OVA) allowed for better tumor growth control [61]. Muliaditan et al. used tin mesoporphyrin in combination with 5-fluorouracil to reverse immunosuppression by myeloid-derived HO-1 activity in a spontaneous breast cancer mouse model [62]. Additionally, immunological tumor growth control was superior compared to immune checkpoint inhibition, possibly due to enhanced CD8+ T cell effector function [62]. Therefore, targeting HO-1 with FMD might be a promising strategy to reduce immunosuppression and enhance antitumor immunity [4,26], considering these results.

### 2.3. Clinical Studies on Fasting Combined with Cancer Treatment

Clinical trials have shown beneficial effects of STF in cancer treatment, such as reduced increase in DNA-damage in PBMCs after chemotherapy administration [8,9,11,64,65,66,67] and reduction of clinical side effects and possibly improved quality of life (QoL), as shown in Table 2 [8,65]. It is important to note that these studies are small and should be validated by larger trials.

At first, a case series by Safdie et al. reported on patients (*n* = 10) diagnosed with different types of cancers, who water-only fasted for 48–140 h prior to chemotherapy and/or 5–56 h following chemotherapy [9]. Patients were their own control, as some chemotherapy cycles were accompanied by fasting and others were not. Six out of ten patients reported fewer chemotherapeutic side effects, such as, less fatigue, weakness, and gastrointestinal issues, when fasted. One patient experienced reduced myelosuppression. These results suggest that STF can relieve chemotherapy induced toxicity.

Similar results were reported by Dorff et al. in another feasibility trial. Twenty patients with diverse malignancies, divided into three groups, water-only fasted for (1) 24 h or (2) 48 h prior, or (3) 48 h prior to and 24 h (72 h total) after platinum-based chemotherapy [66]. Moreover, ≥48 h of fasting tended to be associated with reduced DNA damage in leukocytes (*p* = 0.08) assessed by COMET assay. There was also a non-significant trend towards less grade 3 or 4 neutropenia in the 48 and 72 h cohorts compared to the 24 h fasting cohort (*p* = 0.17).

Moreover, Bauersfeld et al. found indications that water-only fasting for 60 h around chemotherapy administration was associated with a higher QoL score and experiencing less fatigue in 34 patients with gynecological cancer [65]. In this small crossover trial, patients were randomized to fast during the first half of chemotherapy cycles, followed by a regular diet during the second half, or vice versa (NCT01954836).

A randomized pilot study by de Groot et al. (NCT01304251) included 13 early stage breast cancer patients, half of whom water-only fasted for 24 h before and after chemotherapy [67]. Results suggested that water-only fasting might diminish the induction and/or speed up the recovery of chemotherapy-induced DNA damage in healthy PBMCs, as assessed by y-H2AX intensity in CD45+ and CD3+ T lymphocytes [66]. These results indicate that fasting might protect healthy cells from chemotherapy-induced DNA damage [66,67].

This inference is supported by the results of our randomized phase 2 DIRECT trial (DIetary REstriction as an adjunct to neoadjuvant ChemoTherapy for HER2-negative breast cancer), which included randomized patients between FMD and regular diets 3 days before and on the day of neoadjuvant chemotherapy, showing that FMD reduced DNA damage in T-lymphocytes after chemotherapy administration as compared to the regular diet arm (*p* = 0.045) [8]. Although the study had to be aborted halfway for practical and statistical reasons, a complete or partial radiological response of the tumor occurred more often in patients receiving FMD (OR 3.168; CI 1.062–9.446; *p* = 0.039). Furthermore, the per protocol analysis of the pathological response revealed that a 90–100% tumor cell loss was more likely to occur in the FMD-group (OR 4.109; 95% CI 1.297–13.02; *p* = 0.016). We observed no difference in grade 3 or 4 toxicity between the two treatment arms, although it is worth noting that dexamethasone to alleviate toxicity was omitted in the FMD arm. Finally, QoL and illness perception domains appeared to improve in patients receiving FMD [68]. To build upon these results, we are preparing the DIRECT2 trial to validate results in a larger patient group (grants awarded).

Recently, Vernieri et al. published the results of an observational trial evaluating the impact of 5-day FMD cycles every 3–4 weeks in patients (*n* = 101) with various types of cancer [11]. A flow cytometry analysis of PBMCs (*n* = 38) revealed a reduction of peripheral MDSCs subsets (CD14+ HLA-DRneg, CD14+ programmed cell death protein 1 ligand+ (PD-L1) and CD15+) and CD3+ CD25+ T cells after each FMD cycle. Furthermore FMD induced an increase in cytolytic natural killer (NK) cell activity as well as a boost in CD4+ and CD8+ TIL activity defined by programmed cell death protein 1 (PD-1) and CD69 co-expression. These observations were accompanied by altered intra-tumoral immune composition, more specifically numbers of CD8+ T cells, and activated DCs and NK cells were increased, as well as by systemically interferon gamma activating immune signatures that are generally associated with better prognosis in cancer. In a subset of 18 breast cancer patients undergoing surgery, the resection sample showed a significant decline in IGF-1R staining and increased CD8+ TILs compared to the paired pre-FMD biopsy. Moreover, RNA-seq data analyses of transcriptomic signatures suggested an increase in M1 type macrophages, whereas M2 macrophages did not change significantly after FMD. These findings were supported by immunohistochemistry of CD8+ TILs, CD68+ cells, Perforin 1, granzyme B, IGF-1R, and phospho-IGF-1R. In concert, these data support the theory that fasting boosts antitumor immunity and calls for further investigation of the antitumor efficacy of fasting in larger randomized clinical trials.

### 2.4. Fasting Can Synergize with Other Cancer Therapies, including Endocrine, Radiation, and Immunotherapy

A number of translational studies combined fasting with cancer treatment modalities other than chemotherapy, such as endocrine therapy, radiation therapy, immunotherapy, and targeted therapy [10,69,70,71].

Caffa et al. hypothesized that fasting could support endocrine therapy, since 75% of all breast cancers are hormone receptor positive (HR+) and IGF-1 and insulin signaling through the PI3K-Akt-mTOR axis enhances the activity of this hormone receptor resulting in endocrine resistance [69]. The authors demonstrated that FMD boosts the effectiveness of fulvestrant and tamoxifen in an HR+ breast cancer mouse model, via upregulation of early growth response 1 (EGR1), which coincided with decreased PI3K-Akt-mTOR signaling. Furthermore, the authors showed that cyclin-dependent kinase 4/6 inhibitor palbociclib combined with fulvestrant and periodic FMD cycles led to prolonged tumor control and reversed acquired drug resistance in vivo (*n* = 18). These observations were validated in patients with HR+ breast cancer (*n* = 36) who received a variety of endocrine therapies. Lowered levels of circulating insulin, IGF-1 and leptin were measured after an average of 5–6 FMD cycles of 5 days in a feasibility trial. These metabolic changes persisted for 1–3 weeks after FMD for IGF-1 and leptin. Four patients received endocrine therapy and palbociclib combined with FMD and showed promising results, such as longer progression free survival [69].

Manukian et al. studied the immunomodulatory effects of caloric restriction in triple negative breast cancer in mice treated with radiation therapy (RT) [70]. While RT promoted ICD, it also increased the percentage of CD4+ CD25+ Foxp3+ T cells among TIL. In contrast CD8+ T cells to Treg ratio in the TILs was increased four-fold in calorie-restricted mice (1.11% in RT group vs. 4.2% in calorie-restricted + RT group; *p* < 0.002). Caloric restriction induced tumor control was at least partially mediated by CD8+ T cells, as their anti-CD8-antibody-induced depletion during caloric restriction resulted in rapid tumor outgrowth and decreased median survival. Moreover, PD-1 expression was increased in tumor-infiltrating CD3+ CD8+ T cells immunofluorescence analysis in calorie-restricted mice compared to mice fed ad libitum, which likely reflects enhanced activation of T cells. Indeed, tumor immunity was boosted and Treg numbers declined in mice receiving RT accompanied by calorie restriction [70]. Notably, early stage breast cancer patients undergoing RT while receiving 75% of baseline diet calories (*n* = 28) were reported to show signs of reduced systemic immunosuppression, defined as decreased serum levels of IL2-receptor γ, IL-10 receptor β, TGF-β2, and TGF-β3, when compared to patients treated with RT only (*n* = 10) [70].

An experimental study in non-small cell lung cancer with several syngeneic tumor models demonstrated that short-term starvation for 48–72 h sensitizes tumor cells to immune checkpoint blockade with antibodies against PD-1 [10]. Short-term starvation and anti-PD-1 immunotherapy inhibited progression and metastasis of breast- and melanoma cancer. This antitumor effect was dependent on CD8+ cells. Additionally, the treatment combination increased intra-tumoral CD8+/Treg ratio and boosted tumor-specific immunity. Conversely, the authors report that anti-PD-1/ therapeutic resistance is associated with high plasma levels of IGF-1 or high tumor IGF-1 receptor expression in non-small-cell lung cancer patients, which by extension suggests that lowering IGF-1 through STF could be beneficial. In another preclinical mouse model, Lévesque et al. employed a triple strategy of ICD-inducing chemotherapy (mitoxantrone and oxaliplatin) combined with fasting or calorie restriction mimetics (hydroxycitrate or spermidine) and immune checkpoint inhibitors targeting the interaction between PD-1 and its ligand [71]. This strategy cured most of the tumor-bearing mice by establishing T cell-dependent control of tumor growth, while ICD-inducing chemotherapy without fasting or calorie restriction mimetics sensitized the tumor only modestly to treatment with PD-1 blockade alone [71].

These translational results demonstrate the synergistic potential of combining chemoimmunotherapy with fasting regimens. Taken together with the evidence supporting the immunomodulating effects of STF, clinical trials investigating the combination of fasting or FMDs with (chemo-) immunotherapy or targeted therapy are warranted.

## 3. Discussion

Preclinical studies showed that STF as an adjunct to various cancer therapies can potentially improve antitumor immunity, by decreasing immunosuppression and enhancing CD8+ cytotoxicity. Additionally, STF stimulates hematopoietic stem cell regeneration and accumulation of memory T cells in the bone marrow compartment in drug-treated cancer mouse models, which was associated with increased numbers of naïve T cells in some studies [4,38,72]. Moreover, a fasting-induced decrease in tumor-induced immunosuppression was associated with better prognosis in vivo. These findings make a strong case for synergism between fasting and other cancer therapies. Several translational studies have successfully explored the synergistic potential of combinations with chemoimmunotherapy, targeted-, radiation-, and endocrine therapy. Future research can build on these cornerstones.

A beneficial impact of STF on adverse effects of chemotherapy has been observed in several clinical trials [9,65]. In keeping with these observations, dexamethasone omission did not increase the side effects in breast cancer patients receiving FMD in the DIRECT trial, despite the toxicity of anticancer agents [8]. In addition, PBMCs had less chemotherapy-induced DNA damage in these patients, suggesting that FMD protected against chemotoxicity [8]. These findings support the DSR paradigm, which predicts that healthy cells and tissues are better protected against chemotoxicity by STF [6,19]. Finally, our DIRECT trial showed some beneficial effect of adding FMD to neoadjuvant chemotherapy on clinical response [8]. Nevertheless, further research is needed to validate the preclinical findings, as well as the preliminary positive clinical effects of STF on anticancer therapy.

Compliance remains a challenge for fasting in clinical practice. Variations in dietary options and taste, as well as support by a nutritionist or dietician could help. Future studies should incorporate these and other measures to optimize compliance. Patients will be even more motivated to adhere to the dietary rules once evidence of efficacy has been confirmed as it did preclinically. Lastly, a better understanding of STF and FMD on the immune system and antitumor immunity is required. We are currently initiating the FIND study (the effect of a Fasting mimickINg Diet on the immune system: an exploratory study; NCT04833439) to examine the immunomodulatory effects of FMD in healthy individuals in depth, using the RNA NanoString analysis on PBMCs collected before and after two cycles of a 4-day FMD. The outcomes of this small explorative trial will help to better understand the direction of the immunomodulating effects by FMD, which in turn will aid in the setup of future clinical trials, such as the DIRECT-2 trial (expected recruiting in 2022).

## 4. Conclusions

In pre-clinical models, STF can support antitumor immunity by increasing immunogenicity and relieving immunosuppression. This evidence warrants clinical trials investigating the combination of fasting or STF with chemoimmunotherapy and other cancer treatment modalities, examining STF effects on the immune system. In the future, large phase 3 clinical trials are essential to evaluate if and for which types of cancer therapy STF enhances the therapeutic efficacy.

## 5. Key Messages

- Preclinical evidence shows that STF can reduce adverse events and improve the antitumor effects of chemotherapy.
- Preclinical evidence demonstrates that STF can potentiate antitumor immunity, in part by enhancing immunogenicity and relieving tumor-induced immunosuppression.
- Clinical trials show that short-term fasting around chemotherapy is safe in a select group of fit patients and may increase the effectiveness of chemotherapy.
- (Pre-)clinical evidence suggests fasting regimens might alleviate immunosuppressive effects of chemotherapy.
- More studies are required to confirm that STF boosts the efficacy of cancer therapy, such as chemotherapy and immunotherapy, by enhancing tumor immunity.

## Figures and Tables

**Table 1:** Overview of preclinical studies in vivo reporting immunomodulatory effects of STF in the context of cancer treatment.

Author	Mouse Model	Treatment	Outcome, Findings
Pietrocola et al., 2016 [3]	Autophagy competent TC-1 non-small cell lung cancer	Hydroxycitrate + MTX or OX	Improved tumor growth control mediated by Treg depletion; autophagy required
	Colorectal cancer	Hydroxycitrate + MTX or OX	Improved tumor growth control mediated by Treg depletion; autophagy required
	MCA205 fibrosarcoma	Hydroxycitrate + MTX or OX Hydroxycitrate + cisplatin 48-h STF	Improved tumor growth control mediated by Treg depletion; autophagy required Tumor growth control not improved; autophagy induction similar to hydroxycitrate
Di Biase et al., 2016 [4]	4T1 breast cancer, B16 melanoma	2–3 cycles, 4-day FMD every 2 weeks + DXR or CP	Delayed tumor progression; intratumoral Treg reduction, HO-1 dependent; increased CD8+ TILs
			Increased CLP in bone marrow, increased circulating naïve T cells
Takakuwa et al., 2019 [37]	C57Bl/6 mice	48-h STF	Increased naïve CD4 and CD8 T cells in bone marrow
Collins et al., 2019 [38]	C57Bl/6 mice (several types), with B16 melanoma cell line	Calorie reduction 50% for 6 weeks	Central accumulation memory T cells associated with ↑tumor immunity and survival benefit; transient peripheral ↓CD4 & CD8 T cells (1 w); central increase CD4 and CD8 T cells (at 3 w)
Cheng et al., 2015 [39]	C57BL/6 J mice	48–120 h STF around CP administration for 6 cycles in 12–14 days	Hematopoietic stem cell regeneration, IGF-1/PKA dependent; reduced chemotherapy induced mortality; reduced DNA damage in bone marrow cells
Pomatto-Watson et al., 2021 [40]	4T1 breast cancer	4-day FMD, 2 cycles in 28 days	Reduced MDSCs frequency in spleen; intratumoral MDSC frequency; increased T cell to MDSC ratio in PBMC
Sun et al., 2017 [36]	Colorectal cancer, in vivo	Alternate day fasting for 2 weeks	Tumor growth inhibition; less M2 polarization of macrophages
	CT26 and RAW264.7 cells, in vitro		Mechanism in vitro: CD73 reduction, blunted adenosine release into ECM

MTX mitoxantrone, OX oxaliplatin, DXR doxorubicin, CP cyclophosphamide, STF short-term fasting, FMD fasting mimicking diet, HO-1 heme oxygenase-1, Treg regulatory T cell, TIL tumor infiltrating lymphocytes, CLP common lymphoid progenitor, IGF-1 insulin growth factor 1, PKA protein kinase A, MDSC myeloid derived suppressor cells, PBMC peripheral blood mononuclear cells, ECM extracellular matrix.

**Table 2:** Overview of clinical trials observing potential immunomodulatory effects of STF in cancer treatment.

Author, Location	Human Participant	Treatment	Outcome
Safdie et al., 2009, USC, USA [9]	*n* = 10, with different malignancies	Variable, water-only fasting 48–140 h prior to and/or 5–56 h after	Fewer side effects (self-reported)
Dorff et al., 2016, USC, USA (NCT00936364) [66]	*n* = 20, with different malignancies	Platinum-based chemotherapy with 24, 48, or 72 h of water-only fasting	Reduced DNA damage in leukocytes; trend towards less grade 3 or 4 neutropenia
Bauersfeld et al., 2018, Charite University, Germany (NCT01954836) [65]	*n* = 34, women with gynecological cancer	Water-only fasting 60 h around chemotherapy administration	Higher QoL score association
de Groot et al. LUMC, the Netherlands (NCT01304251) [67]	*n* = 13, women with breast cancer (HER2-negative)	TAC CT and 48 h STF	Reduced DNA-damage in PBMCs
de Groot et al., 2020, LUMC, the Netherlands (NCT02126449) [8]	*n* = 131, women with breast cancer (HER2-negative)	Randomized 4-day FMD or regular diet around neoadjuvant CT, maximum of 8 cycles	Increased rate of CR or PR in ITT; increased rate of pathological response per protocol; reduced DNA-damage in PBMCs; QoL non-significant improved in FMD arm; similar grade 3–4 toxicity between arms
Vernieri et al., 2022, University of Milan, Italy (NCT03340935, NCT03454282) [11]	*n* = 101, with different malignancies	5-day FMD every 3–4 weeks	Reduction peripheral MDSCs (*n* = 38); boost CD4 and CD8 T cell peripheral; increased NK cytotoxic activity; intratumoral: ↑CD8 TIL, ↑DCs, ↑NKs; systemic increased IFNγ
	Breast cancer subgroup (*n* = 18)		Intratumoral: ↑M1 macrophage

USC University of Southern California, LUMC Leiden University Medical Center, HER2 human epidermal growth factor receptor 2, CT chemotherapy, QoL quality of life, TAC docetaxel/doxorubicin/cyclophosphamide, STF short-term fasting, PBMC peripheral blood mononuclear cell, CR complete response (radiological), PR partial response (radiological), MDSC myeloid derived suppressor cell, NK natural killer cell, DC dendritic cells, IFNγ interferon gamma.

## Supplementary Materials

The following supporting information can be downloaded at: https://www.mdpi.com/article/10.3390/cancers14061390/s1, File S1: Search strategy for PubMed Database.

## Abbreviations

AKT, Akt	protein kinase B
AMPK	adenosine monophosphate-activated protein kinase
ATP	adenosine 5′-triphosphate
cAMP-PKA	cyclic adenosine monophosphate protein kinase A
CLP	common lymphoid progenitor
CP	cyclophosphamide
CR	complete response
CT	chemotherapy
DC	dendritic cells
DSR	differential stress resistance
DXR	doxorubicin
ECM	extracellular matrix
EGR1	early growth response 1
FMD	fasting mimicking diet
G-CSF	granulocyte colony-stimulating factor
HER2	human epidermal growth factor receptor 2
HO-1	heme oxygenase-1
HR+	hormone receptor positive
ICD	immunogenic cell death
IFNγ	interferon gamma
IGF-1	insulin growth factor 1
IGF1R	insulin growth factor 1 receptor
M-CSF	macrophage colony-stimulating factor
MDSC	myeloid derived suppressor cells
mTOR	mammalian target of rapamycin
MTX	mitoxantrone
NK	natural killer cell
OX	oxaliplatin
PBMC	peripheral blood mononuclear cell
PD1	programmed cell death protein 1
PD-L1	programmed cell death protein 1 ligand
PI3K	phosphoinositide 3-kinase
PR	partial response
QoL	quality of life
ROS	reactive oxygen species
RT	radiation therapy
STF	short-term fasting
TAC	docetaxel/doxorubicin/cyclophosphamide
TAM	tumor associated macrophages
TIL	tumor infiltrating lymphocyte
Tregs	regulatory T cells
