# Supplementary material for: Short-Term Fasting Synergizes with Solid Cancer Therapy by Boosting Antitumor Immunity
Source: Cancers (Basel). 2022 Mar 9;14(6):1390. doi: 10.3390/cancers14061390 (PMC8946179; doi:10.3390/cancers14061390)
Supplement: Supplementary file 1 [file cancers-14-01390-s001.zip › cancers-1618373-supplementary.pdf]

cancers

MDPI

Review

Short-Term Fasting Synergizes with Solid Cancer Therapy by Boosting Antitumor Immunity

Nadia de Gruil [1], Hanno Pijl [2], Sjoerd H. van der Burg [1] and Judith R. Kroep [1,*]

Supplementary File S1. Search strategy for Pubmed Database

("Fasting"[Mesh] OR "Caloric Restriction"[Mesh] OR "Low-Calorie Diet"[tiab] OR "Low Calorie Diet"[tiab] OR "Low-Calorie Diets"[tiab] OR "short-term fast "[tiab] OR "short-term fasting"[tiab] OR "short term fasting"[tiab] OR "Fast-mimicking diet*"[tiab] OR "fasting-mimicking diet"[tiab] OR "dietary restriction"[tiab] OR "intermittent fasting"[tiab]) AND ("Immune System Phenomena"[Mesh] OR "Immune System Phenomena"[tiab] OR "Immune system"[tiab] OR "Immunomodulation"[Mesh] OR "Immunomodulation"[tiab] OR "Immunomodulations"[tiab] OR " Immunomodulatory Therapy"[tiab] OR " Immunomodulatory Therapies"[tiab] OR "Lymphocytes, Tumor-Infiltrating"[Mesh] OR "Tumor Infiltrating Lymphocyte"[tiab] OR "Tumor Infiltrating Lymphocytes"[tiab] OR "Tumor-Infiltrating Lymphocytes"[tiab] OR "Tumor-Infiltrating Lymphocyte"[tiab] OR "Tumor-Derived Activated Cells"[tiab] OR "Tumor Derived Activated Cells"[tiab] OR "Tumor-Derived Activated Cell"[tiab] OR " Tumor Derived Activated Cell"[tiab] OR "T-Lymphocytes"[Mesh] OR "T-Lymphocytes"[tiab] OR "T-Lymphocyte"[tiab] OR "CD4"[tiab] OR "CD-4"[tiab] OR "CD8"[tiab] OR "CD-8"[tiab]) AND ("Neoplasms"[Mesh] OR "Neoplasm"[tw] OR "Neoplasms"[tw] OR "Tumors"[tw] OR "Tumor"[tw] OR "Tumours"[tw] OR "Tumour"[tw] OR "Cancer"[tw] OR "Cancers"[tw] OR "malignancy"[tw] OR "malignancies"[tw] OR "malignant"[tw] OR "oncology"[tw] OR "oncologic"[tw] OR "oncological"[tw] OR "carcinoma"[tw] OR "carcinomas"[tw] OR "cancerous"[tw] OR "chemotherapy"[tw] OR "Antineoplastic Agents"[Mesh] OR "antitumor"[tw] OR "antitumour"[tw] OR "anti-tumor"[tw] OR "anti-tumour"[tw] OR "Medical Oncology"[Mesh])
